# Continuously Reinforced Polymeric Composite for Additive Manufacturing—Development and Efficiency Analysis

**DOI:** 10.3390/polym14173471

**Published:** 2022-08-25

**Authors:** Arvydas Rimkus, Mahmoud M. Farh, Viktor Gribniak

**Affiliations:** 1 Laboratory of Innovative Building Structures, Vilnius Gediminas Technical University (VILNIUS TECH), LT-10223 Vilnius, Lithuania; 2 Department of Steel and Composite Structures, Vilnius Gediminas Technical University (VILNIUS TECH), LT-10223 Vilnius, Lithuania

**Keywords:** additive manufacturing, polylactic acid, aramid fibers, continuous reinforcement, recycling, tensile tests

## Abstract

Additive manufacturing (AM) is a rapidly growing technology, referring to a 3D design process by which digital data builds a physical object in layers by depositing the printed material. The AM has evolved in the aviation, automotive, and medical industries. The AM development for fiber-reinforced composites is the point of current interest, with most research focused on using short fibers. However, notwithstanding particular technological complexities, continuous filaments have superior tensile properties compared to short fibers. Therefore, this manuscript develops an adaptive continuous reinforcement approach for AM based on polymeric material extrusion (ME) technology. It combines the raw material production process, including the ability to vary constituents (e.g., filament materials, reinforcement percentage, and recycled plastic replacement ratio), and the reinforcement efficiency analysis regarding the experimentally verified numerical model. The literature review has identified compatible materials for ensuring sustainable and high-performance plastic composites reinforced with continuous fibers. In addition, it identified the applicability of recycled polymers in developing ME processes. Thus, the study includes an experimental program to investigate the mechanical performance of 3D printed samples (polylactic acid, PLA, matrix reinforced with continuous aramid filament) through a tensile test. Recycled polymer replaced 40% of the virgin PLA. The test results do not demonstrate the recycled polymer’s negative effect on the mechanical performance of the printed samples. Moreover, the recycled material reduced the PLA cost by almost twice. However, together with the potential efficiency of the developed adaptive manufacturing technology, the mechanical characteristics of the printed material revealed room for printing technology improvement, including the aligned reinforcement distribution in the printed product and printing parameters’ setup.

## 1. Introduction

### 1.1. Research Rationale

Fiber-reinforced polymer (FRP) composites are the point of interest in industrial applications because of their high strength-to-weight ratio, magnetic transparency, and corrosion resistance [1]. Continuous fibers are typical FRP constituents [2,3]. Following the literature classification, the FRP manufacturing process can include resin transfer molding [4], manual layup [5], automated tape laying [6], spray-up [7], automated fiber placement [8], pultrusion [9], and filament winding [10]. Unfortunately, molding technologies limit product formability, making complex geometries time-consuming and costly, complicating the FRP innovation progress.

The design flexibility, automated fabrication, and the need for sustainable and low-cost products caused the development of additive manufacturing (AM) for producing FRP components [11]. The AM technologies refer to the fabrication processes by which digital 3D design data builds a physical part in layers by depositing material, extending the FRP engineering horizon for complex geometries without elaborate setups (e.g., automated fiber placement [12] and compression molding [13]), which increased fabrication performance and reduced production costs [14].

This study considers the development possibility of an adaptive continuous reinforcement technology for AM employing polymeric material extrusion (ME) processes. It combines raw material production, including the ability to vary constituents (filament materials, reinforcement ratio, recycled polymers, etc.), and reinforcement efficiency analysis based on experimentally verified numerical modeling [2,3]. This complex methodology would ensure a flexible fabrication of reinforced components for scientific purposes at the current state of the research. In addition, the flexibility and relatively low costs of ME technologies, which do not require post-processing typical for other mainstream AM processes such as stereolithography and selective laser sintering [15], motivate the research idea, making it attractive for industrial applications.

A wide range of printable materials, including polymers and recycled polymer-based products, can be employed through AM. Colorado et al. [16] separated the recycling technologies used in AM into four main categories depending on the raw material: metals, ceramics, plastics, and composites. The plastic recycling process is the most complex among the above categories. Different plastics’ combination reduces the resultant mechanical properties because of the interfacial adhesion defects. Furthermore, it decreases the mixture entropy and causes phase separation [17], and, therefore, the raw materials must be nearly identical, ensuring an efficient product. Two main methods, mechanical and chemical recycling, allow the reuse of plastic waste and thermoplastic materials’ remains. The recycled plastics are applicable after tailoring the mechanical properties to satisfy the AM technology requirements.

A filament developed through the extrusion process is the feedstock material in the ME-based AM. This technology does not require post-processing and defines the open-source base of relatively cheap materials [15,16]. In addition, the AM is flexible to control fiber orientation and position, changing fiber volume fraction and producing functionally graded reinforced structures [12]. Various reinforcement approaches, employing powder, short and long fibers, make ME an efficient alternative to other mainstream AM processes such as stereolithography and selective laser sintering. The following section substantiates the choice of the materials in this study for ensuring sustainable and high-performance plastic composites and discusses the possibility of using recycled polymers.

### 1.2. Continuous Reinforcement and Recycling PLA for AM

Typically, AM employs the material layered depositing process when digital 3D design data forms a physical component. This definition comes from the ASTM committee on AM technologies [18]. Despite the common practice of considering the “3D printing” term synonym for AM, it describes a separate process. In other words, the rapid printing or prototyping processes do not adequately describe AM technologies. The continuous material addition process more accurately describes the AM ideology, distinguishing it from the conventional manufacturing methods based on material removal.

This study focuses on material extrusion (ME) technology suitable for continuous reinforcing. It dispenses fused material in filaments or pastes/liquids form through a nozzle [18]. The relatively low costs and simple operation make this AM technology widely used. The production process involves thermoplastic extrusion, typically in the filament form, in a layer-by-layer deposition dispensing through nozzle molten thermoplastic onto a build platform. The relative nozzle motion regarding the platform allows the building of curvilinear layers and complex geometries from a bottom-up manner [19,20]. Nowadays, most desktop 3D printers employ this technology because of low-cost feedstock and simple operating conditions; the production process can use various thermoplastics similar to injection molding, making it flexible for industrial purposes [15].

Reinforcement improves the AM materials’ mechanical performance, durability, thermal, and electrical properties. Different fiber types and sizes were utilized for that purpose. However, most of research focused on using short fiber reinforcements because of the manufacturing technology’s simplicity compared with the continuous filaments. The literature reports the application examples of single-walled carbon nanotubes (SWCNT) [21,22], multi-walled carbon nanotubes [23], vapor-grown carbon fibers [21], graphene [24], micrometer-sized copper powders [25], and millimeter-chopped fibers of thermotropic liquid crystalline polymers [26], glass [27], and carbon fibers [28,29]. For example, adding 13wt% millimeter-sized carbon fibers increased in-plane tensile strength and elasticity modulus of FRP by 250% and 400%, respectively [29]; using 10wt% nanoscale SWCNT demonstrated an increase of 39% and 61% to the corresponding parameters [21].

There also has been a growing interest in developing functionalized materials with a wide range of properties, such as thermal [30], electrical conductivity [31], piezoelectric [32], and electric transparency [33]. The ME apparatus does not require complex modifications to produce parts reinforced with short fibers. However, processing continuous fiber-reinforced composites involves software and hardware adjustments, e.g., nozzles with fiber feeding ports and cutting trajectories [34]. Still, this study develops an adaptive continuous reinforcement technology for AM, which combines raw material production and reinforcement efficiency analysis based on experimentally verified numerical modeling [2,3]. The considered adaptive methodology ensures a flexible fabrication of reinforced components for scientific purposes at the current state of the research. Thus, this investigation deals with simplified geometry testing specimens to avoid the necessity of developing automated fiber-cutting equipment. On the contrary, the existing printing open-source software was modified in this study to maintain a continuous extruder pathway through all printing processes; manual fiber cutting is only necessary at the end of the printing process. Therefore, the automated cutting procedures remained beyond the scope of the literature review.

Continuous fibers have superior tensile properties compared to short fibers [34]. Carbon, glass, and aramid filaments are the typical continuous reinforcements. Table 1 summarizes the application results of continuous macro-fibers—the centerline of this research.

**Figure 1:**
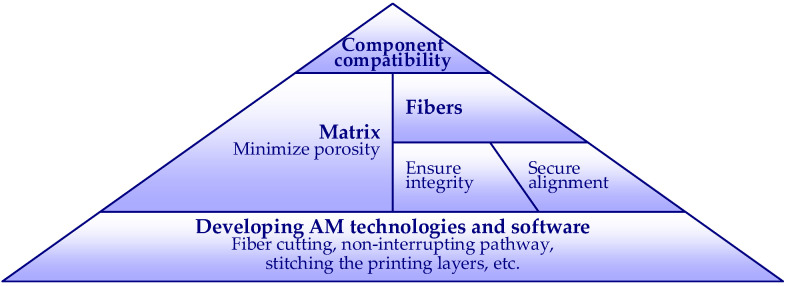
The polymeric components with a continuous reinforcement development structure.

Studies [35,36,37,38,39,40,41,42,43,44,45,46,47] in Table 1 demonstrated the quality and mechanical performance of continuously reinforced polymeric composites manufactured by ME-based AM. Thus, these results helped to describe the development structure of the continuous reinforcement for polymeric components shown in Figure 1. In this scheme, the compatibility condition ensures the composite interaction between the polymeric matrix and fiber reinforcement; the reinforcement integrity allows fibers to absorb the load from the polymeric matrix, which distributes and protects the continuous filaments from mechanical and environmental effects. The interfacial adhesion also prevents delamination failure, securing acceptable mechanical performance of the FRP composite. On the other hand, the fibers’ alignment and homogeneity ensure consistent mechanical properties throughout the printed structure; the fiber distribution control ensures the efficiency of the reinforcement. In addition, minimizing material porosity reduces the heterogeneity of the mechanical properties and improves FRP durability. Further modifying AM technologies (Figure 1) adapts the manufacturing equipment for continuous reinforcement (fiber cutting or separate polymer and filament extrusion) and software development (uninterrupted path printing, 3D pathways layup, stitching the printing layers, etc.).

However, despite the rapid progress of AM technologies, most systems use a limited range of commercial and proprietary resins, which restrict the development of the physical and chemical properties of the products [48]. Thermoplastics are the typical matrix materials for ME. Polylactic acid (PLA) is one of the most investigated thermoplastic materials; it operates at 180–230 °C, and the ME process does not require a heated bed [23].

Furthermore, the PLA components are biodegradable and feasible for recycling. For instance, the articles [49,50,51,52] investigated the mechanical properties of recycled PLA produced in the form of filaments suitable for 3D printing. As a result, the viable PLA for AM and corresponding methodology have been introduced for recycling polymers in ME. The investigation exhibited the potentiality of developing AM samples using recycled PLA; however, the degradation of the polymer mechanical performance resulted from repeated recycling [52].

Comparing the mechanical properties of virgin PLA and recycled PLA (up to three times) demonstrated almost identical strength of the once and twice recycled test specimens regarding the virgin PLA. On the other hand, the third recycling process affected the strength results negatively [50], causing a reduction in the tensile strain rate. In addition, the five-times recycling of PLA increased the tensile modulus and tensile strength but caused a 10% reduction in the ultimate elongation [51].

Tian et al. [42] systematically investigated the recycling and remanufacturing processes of 3D printed PLA carbon fiber (8.9vol%) composite. The continuous carbon fiber reinforced PLA matrix was recycled from the 3D printed components and converted into a PLA-impregnated filament form, representing a feedstock material for 3D printing. The results exhibited an increased flexural strength regarding the reference samples, which was attributed to the fiber–matrix interface enhancement. On the other hand, remanufacturing reduced the flexural modulus from 15 GPa to 13 GPa. The studies [53,54] formulated particular requirements to adapt the recycled PLA properties for 3D printing.

The literature analysis highlighted a broad PLA application in the ME research [23,35,36,38,40,42]. Regarding alternative polymers available on the market, the low thermal shrinkage, low-temperature printability, and recycling possibilities [49,50,51,52] supported the PLA selection for this study. In addition, a continuous aramid filament reinforces the PLA matrix to prevent the polymeric composite’s brittle failure because of the aramid toughness and high strain at the peak load compared to the carbon and glass fibers [40].

Notwithstanding interest in AM technology development, continuous reinforcement layup faces severe limitations discussed above. These restrictions motivated this study to fill this gap considering the experimental program implemented in the Laboratory of Innovative Building Structures at VILNIUS TECH—the 3D printed elements (PLA matrix reinforced with continuous aramid filament) are the tensile test subject. Recycled polymer replaced 40% of the virgin PLA in the composite.

## 2. Experimental Program

The experimental program consists of two stages. The first stage investigates the mechanical properties of alternative PLA materials and verifies the possibility of partial replacement of the source material with recycled plastic. The mechanical performance of ME components produced from the partially recycled PLA reinforced with continuous aramid fiber is analyzed in the second stage. The test campaign contains 28 tensile test specimens—18 tested in the first stage had no reinforcement; the remaining 10 reinforced polymeric samples belong to the second testing stage. The ASTM D638-14 [55] and D3039 [56] standards have determined the samples’ geometry and test procedure. All elements were produced with a Prusa i3 MK3 printer (Prusa Research, Prague, Czech Republic) applying PrusaSlicer 2.3.3 slicing software (Prusa Research, Prague, Czech Republic).

**Figure 2:**
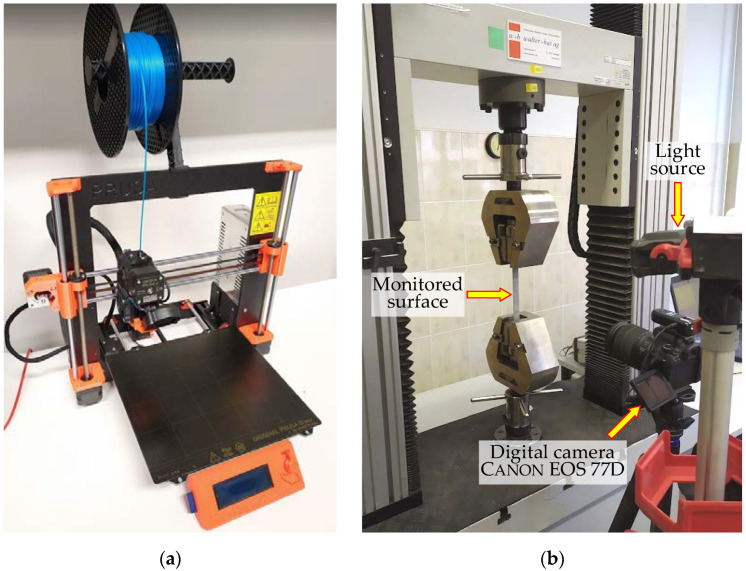
Preparing test specimens: (**a**) A Prusa i3 MK3 printer; (**b**) Tensile test setup.

The tension tests were carried out using a 100 kN electromechanical apparatus LFM-100 (Walter + Bai ag, Löhningen, Switzerland) with a 2 mm/min loading rate; a 100 kN load cell measured the load. Figure 2 shows the Prusa i3 MK3 printer and the testing setup.

A digital image correlation (DIC) system monitored the relative displacements of the specimen surface (Figure 2b). The GOM Correlate software (Gom Metrology, Braunschweig, Germany) mapped the strain distribution. The spray paint ensured a high-contrast random pattern to facilitate the software tracking of the movement tensors of the pixels recognized on the surface’s digital images. This technique enables monitoring relative displacements of arbitrarily set pixels after the physical tests [2,3]. A digital single-lens reflex camera Canon EOS 77D SLR (Canon Inc., Tokyo, Japan) with 18–135 mm Canon EF-S lens placed on a tripod at 0.4 m from the monitored surface captured the digital images using the following settings: the exposure time was 1/100 s, the aperture was f/4.0, the focal length was 24 mm, and the sensitivity to light was ISO 100 according to [57]. The 6000 × 4000 px images were captured at the 0.25 kN load increment. A remote-control device allowed for avoiding unexpected movements of the camera.

### 2.1. Tensile Tests of the Printed Polymers

The first testing stage employs the tensile specimens of three different PLA materials. Peinado et al. [58] and Anderson [59] reported that the reuse of multiple (up to 20) times of recycled PLA through the ME process minimally affects the mechanical performance of the printed material. Significant alterations in the mechanical properties were also not identified. These results allowed this study to hypothesize the absence of the negative effect related to the recycled polymer application in ME. Therefore, the locally produced, partly recycled PLA (Inorega Ltd., Rudausiai, Lithuania) is the investigation object to verify the above hypothesis. This material contains 40% of the PLA compound recycled once. The 100% primary PLA from the same producer represents the reference; the comparative analysis also includes the reputable PLA produced in Europe (Prusa, Prague, Czech Republic). The materials in the filament form with a 1.75 mm nominal diameter were used for ME.

**Figure 3:**
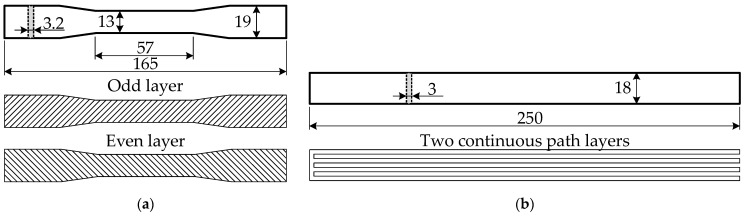
Sample geometry and printing layout (dimensions are in mm): (**a**) Unreinforced specimen; (**b**) Reinforced plate.

**Figure 4:**
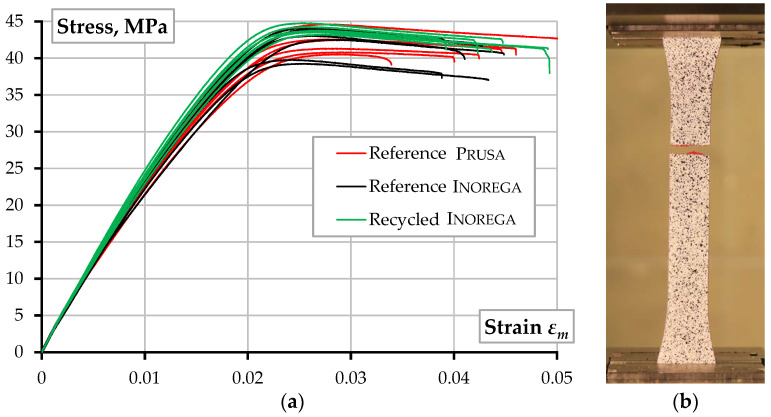
Unreinforced samples: (**a**) Stress-strain diagrams; (**b**) Characteristic failure.

**Figure 5:**
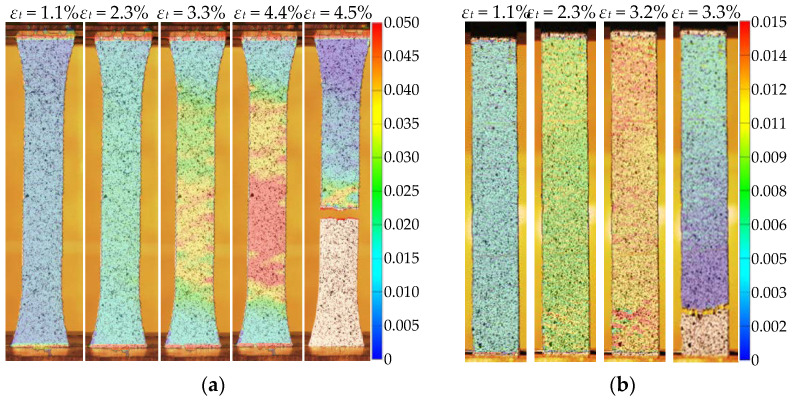
Strain distribution mapped with DIC: (**a**) Unreinforced specimen; (**b**) Reinforced plate.

The unreinforced tensile specimens correspond to Type I by the ASTM standard D638-14 [55]. The printing layout of the dumbbell-shaped test samples (Figure 3a) includes two solid “shells” located at the printing perimeter; the inner part of the specimen was printed in 11 layers with 100% infill oriented at the ±45° angle regarding the loading direction. The infill angle was altered in every layer. The 3D printing was carried out through a 0.4 mm nozzle at a 210 °C temperature and a 28 mm/s speed; the bed temperature was 60 °C. Six specimens of each material were manufactured using the above parameters. The 18 unreinforced samples were tested to compare the mechanical performance of the printed PLA materials. Figure 4 shows the stress-strain diagrams and the characteristic failure mechanisms (the actual cross-section dimensions determined stresses in the polymer). Figure 5a shows the DIC analysis example.

Table 2 identifies the mechanical performance of the tested samples. This table also includes the average values of identical specimens and the corresponding coefficient of variation (CV). These results align with the Prusa manufacturer specified characteristics of such elements: the yield strength *f_y_* = 50.8 MPa; the modulus of elasticity *E_p_* = 2.2 GPa; the elongation at the yielding point *ε_y_* = 2.9%.

In addition, Table 2 and Figure 4 demonstrate that the mechanical characteristics of partially recycled PLA concede to neither the primary material nor alternative PLA by Prusa. The observed result aligns with the findings of Lanzotti et al. [50] and Zhao et al. [51] when multiple recycling (up to three times) caused no significant alteration in the tensile strength of the printed material. Zhao et al. [51] also documented a reduction in the ultimate strain by 10% of fully recycled PLA. The latter effect was not identified in the present tests, possibly because of the partial replacement of the virgin PLA with recycled material. At the same time, the recycled polymer almost twice reduces the final product (raw filament) cost, making the ME technology economically efficient. Furthermore, the 40% PLA replacement with recycled plastic seemingly could improve the mechanical performance and reduce the scatter of the test results (compare the “Reference Inorega” and “Recycled Inorega” results in Table 2). Thus, this outcome identifies the object of further research, and, therefore, further development of the reinforced composite (Section 2.2) employs partial replacement of PLA with recycled plastic (at the 40% ratio).

### 2.2. Tensile Tests of Reinforced PLA

This work develops a continuous reinforcement technology for AM, which combines raw material production and reinforcement efficiency analysis. Thus, in cooperation with Inorega Ltd. (Rudausiai, Lithuania), the continuously reinforced polymeric filament manufacturing equipment was developed by modifying the TD-SJC50 series extruder Tengda (Nanjing Tengda Machinery, Nanjing, China) that comprises a vacuum feeder, hopper dryer, single screw extruder, head mold, heat and cooling tanks, laser gauge of the filament diameter, and haul-off machines. The production unit is equipped with a 50 mm single screw feeder, with a 28/1 length to diameter ratio and a varying diameter extruder; a 15 kW engine allows the production capacity to reach 20 kg/h. The modification was aimed at developing the custom-made dry fiber feeding, impregnation, and centering apparatus incorporated in the head mold of the extruder. After the cooling, the diameter of the reinforced filament was monitored using a laser sensor and adjusted if needed. The target 1.75 mm diameter of the filament was selected after several trials, considering the typical printers’ possibility of using the reinforced filament for ME.

The para-aramid 1K yarn of 12 μm fibers Twaron 1610 dtex (Teijin Aramid, Arnhem, The Netherlands), having a total 0.38 mm diameter, was used for the reinforced PLA filament production in this study. The producer provided the following characteristics of the aramid fiber: density is 1.44–1.45 g/cm^3^; tensile strength is 2.7–3.6 GPa; modulus of elasticity is 60–145 GPa. The modified Tengda extruder distributed the continuous aramid yarn coaxially within the PLA matrix.

This study uses a series of identical reinforced polymeric filaments to produce the composite cross-section. Thus, under the assumption of the manufacturer’s specified yarn parameters and the nominal diameter of the reinforced filament, the following expression describes the fiber volume fraction in the reinforced composite:

(1) $$V_{f}=\frac{A_{f}}{A_{tot}}={\left(\frac{d_{f}}{d_{tot}}\right)}^{2},$$

where *A_f_* and *A_tot_* are the yarn and reinforced filament cross-section areas, *d_f_* and *d_tot_* are the corresponding diameters. In the considered case, the above diameters equal 0.38 mm and 1.75 mm, determining the volume fraction *V_f_* = 4.72%.

The printing software was modified to maintain a continuous extruder pathway through all printing processes; manual fiber cutting is foreseen at only the end of the printing process. In addition, the printing speed and nozzle diameter were adapted for ME of reinforced filament. Thus, the printing process utilizes the conventional Prusa i3 MK3 printer with a 0.8 mm nozzle. The specimens were manufactured at 210 °C and a 10 mm/s speed; the bed temperature was 60 °C. The continuous reinforcement does not allow terminating the printing process. Therefore, the 3D extruder motion and printing flow were tailored to avoid interruption and damaging the pathway. The printing code was customized for that purpose, specifying a constant 26 mm^3^/s volumetric flow of the fibrous PLA composite.

The ASTM D3039 standard [56] has determined the reinforced samples’ dimensions; however, the square plate shape geometry was slightly modified because of the printing pathway limitations. Figure 3b shows the resultant geometry of the 250 × 18 × 3 mm tension samples and the travel path of the extruder. Five PLA specimens reinforced with the aramid fiber were manufactured using the above parameters. Figure 5b shows the DIC example of the reinforced sample—the uniform strains’ distribution is characteristic of the test sample up to failure.

**Figure 6:**
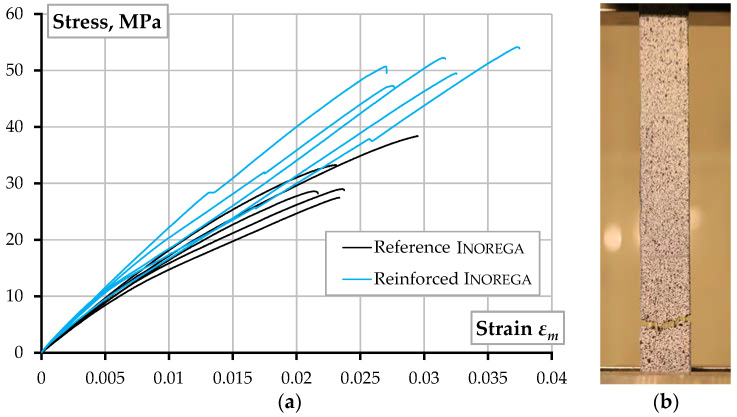
Reinforced samples: (**a**) Stress-strain diagrams; (**b**) Characteristic failure.

For comparison purposes, five additional samples of the same geometry (i.e., the 250 × 18 × 3 mm square plate) were produced without reinforcement using the same printing parameters as the reinforced samples. These test elements defined the reference for estimating the fiber effect on the mechanical performance of the printed material. Table 2 summarizes the test results. The test outcomes indicate the evident improvement of the load-bearing capacity related to the fiber reinforcement—the ultimate load increased by 67% in the reinforced samples. Figure 6 shows the stress-strain diagrams and distinct failure mechanisms of reinforced and unreinforced samples.

Mohammadizadeh and Fidan [60] reported that continuous aramid fiber reinforcement with the 8vol% fiber fraction significantly reduces the ultimate strain and increases the tensile strength of AM polymer composite by approximately three times. The observed tensile stresses (Table 2) do not contradict the literature results [60]. However, the present experimental results do not indicate any ultimate strain reduction in the continuous fiber reinforcement. This might be the cause of low ME quality and poor reinforcement efficiency. These effects are discussed in Section 3.1 and Section 3.2

## 3. Discussion

### 3.1. The ME Quality

**Figure 7:**
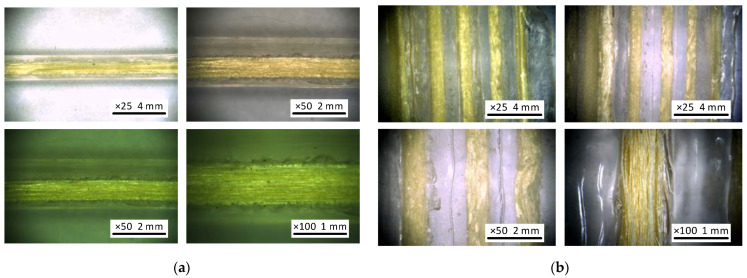
Reinforced samples from transparent PLA: (**a**) Raw material (reinforced PLA filament); (**b**) Printed reinforced sample.

The previous studies [2,3] related the reinforcement efficiency of continuous filaments in an FRP composite with the bonding performance of the reinforcing material in a polymer matrix, and the post-processing, e.g., cutting the test samples, can diminish the adequacy of the mechanical analysis. In addition, the relatively small aramid yarn diameter complicates the analysis of the fiber-bond quality. Therefore, this study includes reinforced specimens produced from transparent PLA from the same producer as the polymeric material used for the mechanical tests (Figure 7a). This solution allows for observing the fiber alignment by an optical microscope without cutting the samples.

The 250 × 18 × 3 mm square-plate specimen (Section 2.2) was printed using transparent PLA to estimate the bond quality and alignment of the continuous fibers. The geometry specified by the ASTM D3039 standard [56] was slightly modified (Figure 3b) because of the printing pathway limitations related to the reinforced filament diameter (1.75 mm). The interfacial connection was observed using a 100× digital microscope DG-3X (Scalar, Tokyo, Japan). Figure 7b shows the fiber–matrix bonding fragment, which demonstrates visible defects of the bond because of the air voids around the fiber. Figure 7b illustrates the duplicated manufacturing result—the aramid yarn first reinforced the extruded PLA filament, and only after that the 3D printer produced the reinforced product. Thus, this methodology accumulates the defects of both manufacturing stages. Therefore, such ME technology raises high-quality requirements for raw materials. This requirement differentiates the considered technology from the ME process when reinforcement yarn is consolidated together with the polymer during the 3D printing process [61].

On the other hand, current manufacturing equipment can control the quality of the reinforced PLA extrusion process. At the same time, the standardized dimensions of the reinforced filament allow for printing reinforced components with slightly customized 3D printers, making the manufacturing process flexible. Remarkably, the customizing process should also account for the printing parameters, i.e., the nozzle diameter, printing speed, and temperature.

**Figure 8:**
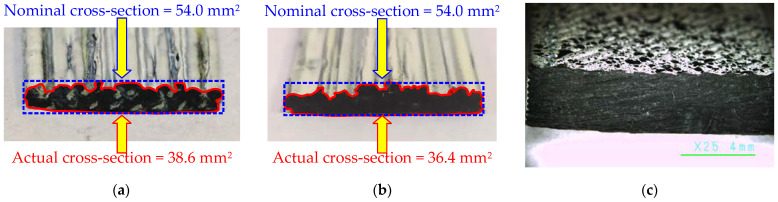
Printing defects: (**a**) Reinforced specimen; (**b**) Unreinforced sample manufactured through 0.8 mm nozzle; (**c**) Unreinforced reference produced through 0.4 mm nozzle (Section 2.1).

Figure 8 exemplifies the printing results of the specimens described in Section 2.2, with printing parameters chosen to ensure the steady printing process. Altering the typical 28 mm/s printing speed through a 0.4 mm nozzle to 10 mm/s 0.8 mm nozzle provided uniform distribution of the reinforcement yarn in the printed structure. However, the customized parameters did not allow for precise geometry—both samples produced, altering the default settings, demonstrate an irregular cross-section shape (Figure 8a,b). The irregularities become apparent regarding the reference sample (Figure 8c). This result indicates sufficient space for further improving the ME parameters.

The partial debonding of the aramid yarn (Figure 7b) could reduce the stiffness of the developed reinforced polymer. Hu et al. [61] stated that the yarn impregnation before the PLA filament extrusion could improve the bond quality. Moreover, the printed specimen’s yarn alignment significantly affects the resultant product’s mechanical performance [62]. Therefore, the filament must be straight without any weaving and misalignments. The ability of the printing equipment to stretch the filament during ME could solve this problem [63].

### 3.2. The Reinforcement Efficiency

Gribniak et al. [2,3] developed a methodology for estimating the reinforcement efficiency when experimentally verified finite element (FE) predictions define the efficiency reference. It employs a simplified FE model based on the smeared reinforcement concept. The model can predict the mechanical resistance (stiffness and load-bearing capacity) under the assumption of experimental elastic moduli of the composite constituents, i.e., the PLA matrix and bare fiber. Following the load-sharing concept [64], the tension (Figure 2) expressed in terms of external load **P** induces two internal forces acting on the aramid fiber **N***_f_* and PLA matrix **N***_p_*:

(2) $$P=N_{f}+N_{p}.$$

The following equation explains the above load components by the average strains *ε*, assuming equality of the considered deformations:

(3) $$\begin{array}{c} E_{m}\cdot A_{m}\cdot \varepsilon_{m}=E_{f}\cdot A_{f}\cdot \varepsilon_{f}+E_{p}\cdot A_{p}\cdot \varepsilon_{p}\\ \varepsilon_{m}=\varepsilon_{f}=\varepsilon_{p} \end{array}\}\Rightarrow E_{m}\cdot A_{m}=E_{f}\cdot A_{f}+E_{p}\cdot A_{p},$$

where *E* and *A* are the elastic modulus and area; the indexes *m*, *f*, and *p* correspond to the composite, aramid fiber, and PLA matrix parameters. The following formulas express the areas in Equation (3) in the volumetric fiber fraction *V_f_* terms:

(4) $$A_{f}=A_{m}\cdot V_{f}\begin{array}{cc} ; & A_{p}=A_{m}\cdot \left(1-V_{f}\right) \end{array}.$$

Based on Equation (4), Equation (3) can be simplified as follows:

(5) $$E_{m}=E_{f}\cdot V_{f}+E_{p}\cdot \left(1-V_{f}\right).$$

Thus, Equation (5) defines the volumetric fiber content expression alternative to Equation (1):

(6) $$V_{f}=\frac{E_{m}-E_{p}}{E_{f}-E_{p}}.$$

Assuming *E_m_* in Equation (6) is equal to the elastic modulus *E_test_* determined through the reinforcement specimen tension tests (Figure 6), the efficient reinforcement fraction, *V_f,ef_*, can be defined as follows:

(7) $$V_{f,ef}=\frac{E_{test}-E_{p}}{E_{f}-E_{p}}.$$

The following coefficient describes the fiber reinforcement efficiency:

(8) $$C_{ef}=V_{f,ef}/V_{f}.$$

Equations (1) and (7) describe the volumetric fractions *V_f_* and *V_f,ef_* in Equation (8).

This study employs the above technique to quantify the efficiency of the developed reinforced composite using non-linear finite element software Atena [65]. The 3D solid finite elements represent the polymer matrix; the fracture mechanic principles describe the PLA failure. A 1D material model coincides with the ME direction and determines the reinforcement; an elastic-brittle constitutive law defines the fiber failure.

**Figure 9:**
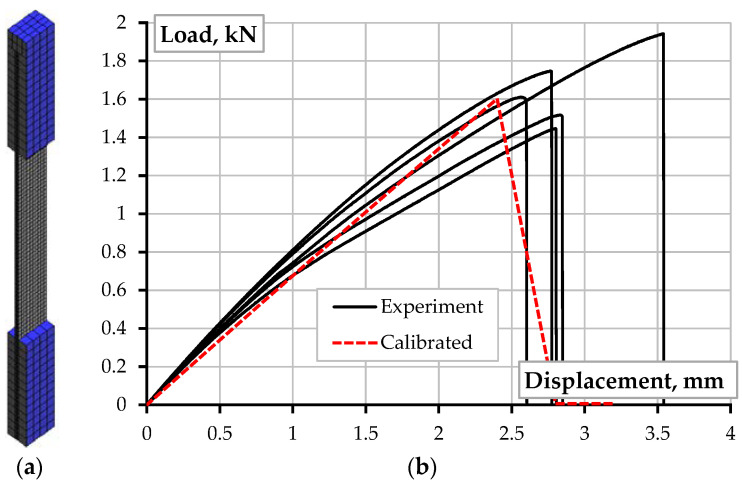
Unreinforced sample: (**a**) FE model; (**b**) Model verification result.

At the first stage, the constitutive law of the PLA material was tailored using the “Reference Inorega” samples (Figure 6a). Figure 9a shows the FE model with isoparametric hexahedral eight-node finite elements, having an average 0.7 mm size and eight integration points (Gauss integration). The FE model approximates the experimental cross-section dimensions (Figure 8b). The modeled steel plates protect the specimen supports that correspond to the physical tests (Figure 2). The isoparametric brick eight-node finite elements with eight integration points discretize the steel plates, assuming the perfect contact to the polymer. The Newton–Raphson iteration procedure controls the deformation problem solution.

Figure 9b shows the model verification results, identifying the parameters: the modulus of elasticity *E_p_* = 2.2 GPa; the tensile strength *f_u_* = 100 MPa; and the fracture energy *G_F_* = 58 N/m. The printing pathway declination from the straight line could explain the more than tripled value of the tailored strength (100 MPa) regarding the maximum stresses estimated in Figure 6a (31.4 MPa). In addition, the structure solidification differences could alter the estimated strength—the results of Figure 6a assume the monolithic structure of the printed PLA. However, Figure 8b shows voids and defects, reducing the cross-section area. Therefore, particular care is recommended in applying the nominal characteristics of polymeric material in the numerical analysis of the manufactured structures because of the ME effect on the produced material structure, which is far from monolithic [66].

**Figure 10:**
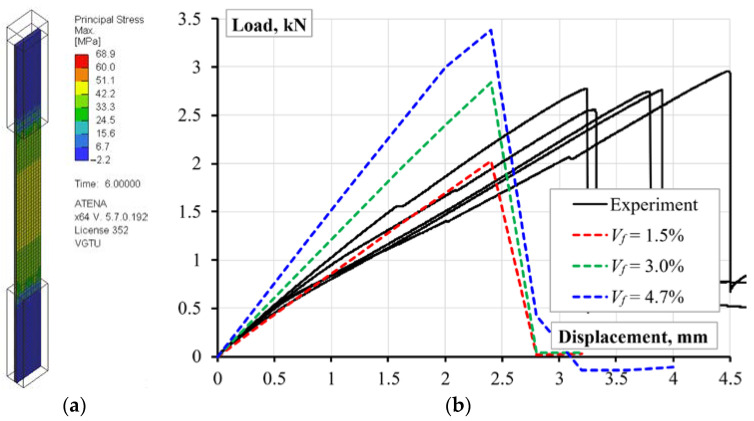
Reinforced sample: (**a**) Predicted stress distribution; (**b**) Efficiency estimation results.

In the second stage, the volumetric fiber content was the simulation variable (all other model parameters remained the same); the simulated geometry approximates the cross-section shown in Figure 8a. Figure 10 shows the simulated stress distribution in the reinforced specimen and compares the numerical prediction and test results. The tension displacement, applied in small increments (0.3 mm), determined the load-bearing capacity of the model. The first model assumes the volume fraction *V_f_* = 4.72%, corresponding to the nominal reinforcement content (Section 2.2): the overestimation of axial stiffness, *EA*, and strength, *f_u_*, is apparent. The fiber content reduction to 3.0% and 1.5% remedies the strength and stiffness predictions, respectively. Thus, Equation (8) defines the 31.8% reinforcement efficiency regarding the axial stiffness; the reinforcement efficiency increases to 63.6% in the load-bearing capacity terms.

The simulation results with the reference fiber content (*V_f_* = 4.72%, Figure 10b) define the space for improving the ME technology for the reinforced composite development. The numerical study outcomes align with the experimental results [60], which demonstrated the continuous fibers’ capacity to control the stiffness of the printed material; increasing fiber content also decreases the ultimate strain of the sample. At the same time, the experimental results reveal beneficial ductility of the developed composite—the deformation corresponding to the maximum resistance of the test specimens more than 1.56 times exceeds the prediction results. This outcome determines the object for further analysis. Moreover, the comparative analysis of the DIC-traced relative displacement maps (Figure 5) reveals the strain distribution uniformity in the reinforced specimens until the polymer fracture, which supports the continuous reinforcement concept.

### 3.3. Polymer Recycling Perspectives

Notwithstanding the recycling limitations [16], existing ME technologies allow efficient re-using of PLA materials. Zhou et al. [53] and Paciorek-Sadowska et al. [54] formulated the recycled PLA adaptation principles for ME. The low-temperature printability, together with a solid scientific background collected in the literature (Section 1.2), stimulates the investigations on recycling PLA waste and makes it a valuable source for further AM development. The published results [58,59] reveal the efficient applicability of re-used PLA (recycled up to 20 times) without noticeable loss of the mechanical performance of the ME components. The present study outcomes also support the hypothesis about the absence of the negative effect of recycled polymers’ application. Table 2 and Figure 4 reveal two important findings:

- The 40% replacement with recycled material does not negatively affect the PLA strength and modulus of elasticity. At the same time, this modification reduced the PLA cost by almost twice, making the ME technology economically efficient.
- The improvement in the mechanical performance, expressed in terms of the strength, elasticity modulus, and ultimate strength, and the scatter reduction of the test outcomes (Table 2), allows this study to postulate the effectiveness of the PLA replacement with recycled plastic, which should be the object of further research.

## 4. Conclusions

This work develops an adaptive continuous reinforcing methodology, which combines the raw material production process and reinforcement efficiency analysis based on experimentally verified finite element (FE) modeling. The test program comprises 28 polymeric 3D printed samples subjected to tension load. The obtained results formed the following conclusions:

- *Continuous reinforcing.* Notwithstanding the additive manufacturing (AM) progress, the literature review identified severe limitations of continuous reinforcement related to the material constituents’ compatibility, the composite structure solidification, fibers’ alignment, and adapting material extrusion (ME) technologies.
- *ME quality.* The case considered in this study exemplifies the duplicated production consequence—the aramid yarn first reinforced the extruded polylactic acid (PLA) filament, i.e., the raw printing material, and a 3D printer produced the reinforced product at the second stage. Such a process accumulates the defects of both manufacturing stages. This feature differentiates the considered technology from the reinforced polymer consolidation processes at the 3D printing stage. However, the raw reinforced filament’s standardized dimensions allow for using typical 3D printers, making the manufacturing process flexible. Further development could focus on developing continuous filament cutting equipment and software for uninterrupted path printing and stitching of the printing layers.
- *Printing layout*. The mechanical performance of the standardized unreinforced PLA samples corresponds to the parameters provided by the manufacturer. However, the printing pathway and parameters customization reduced the printed material’s load-bearing capacity—the average reduction in mechanical strength was equal to 1.40 times. The continuous reinforcement improved the mechanical performance significantly. However, additional tests are necessary to optimize the printing setup.
- *Reinforcement efficiency*. The proposed FE analysis procedure identified the overestimation of axial stiffness and load-bearing capacity—the stiffness and strength terms determine the 31.8% and 63.6% reinforcement efficiency. At the same time, the experimental results reveal beneficial ductility of the developed composite—the deformation corresponding to the maximum resistance of the test specimens more than 1.56 times exceeds the prediction results, determining the further analysis object.
- *Recycling possibility*. The test results do not demonstrate the recycled polymer’s negative effect on of the ME product performance, supporting findings reported in the literature. The 40% PLA replacement with recycled plastic caused an increase in tensile strength (4.2%), elasticity modulus (5.1%), and ultimate strength (7.5%). This outcome defines the object for further analysis. Moreover, the recycled material reduced the PLA cost by almost twice, making the ME technology economically efficient.
- *Further development.* In cooperation with Inorega Ltd. (Lithuania), this study developed the continuously reinforced PLA manufacturing equipment that ensures the raw material production process, including the ability for varying constituents (e.g., reinforcing filaments, reinforcement percentage, and recycled plastics replacement ratio). Thus, the proposed technology ensures a flexible fabrication of reinforced components for scientific purposes. The literature review allowed for selecting compatible raw materials, and the printed structure observation supported the choice. However, the experimentally identified mechanical performance of the printed material indicates room for printing technology improvement, including the aligned reinforcement distribution in the printed product. The prospects for industrial application of the proposed analysis concept also determine the target for further research.

## Figures and Tables

**Table 1:** Continuously reinforced polymeric matrix ME technology products.

Ref.	Fiber/Matrix Type	Outcome	Strength [MPa]
[35]	Carbon fiber (34vol%)/PLA	Flexural strength: 156 MPa	91
[36]	Carbon jute (40–50vol%)/PLA	Elastic modulus: 19.5 GPa	185
[37]	Aramid fiber/Nylon	Elastic modulus (4vol%): 1.78 GPa Elastic modulus (8vol%): 6.92 GPa Elastic modulus (10vol%): 9.0 GPa	31.1 58.8 83.0
[38]	Carbon fiber (1K bundle, 27wt%)/PLA	Flexural strength: 335 MPa Flexural modulus: 30 GPa	–
[39]	Carbon fiber (34.5vol%)/Nylon	Elastic modulus: 35.7 GPa	475
[40]	Aramid fiber (8.6vol%)/PLA	The triple and sextuple increase in tensile modulus and strength regarding unreinforced reference	206
[41]	Carbon fiber (11vol%)/Nylon	Elastic modulus: 7.73 GPa Flexural strength: 250 MPa Flexural modulus: 13.0 GPa	216
	Glass fiber (10vol%)/Nylon	Elastic modulus: 8.42 GPa Flexural strength: 197 MPa Flexural modulus: 4.21 GPa	206
	Aramid fiber (10vol%)/Nylon	Elastic modulus: 4.98 GPa Flexural strength: 126 MPa Flexural modulus: 6.65 GPa	164
[42]	Recycled carbon fiber (8.9vol%)/PLA	Flexural strength: 263 MPa Flexural modulus:13.3 GPa	260
[43]	Glass fiber (54.8wt%)/Polypropylene	Flexural modulus: 13.1 GPa	–
[44]	Carbon fiber/Nylon	Impact strength: 82.3 kJm^2^	–
	Glass fiber/Nylon	Impact strength: 281 kJm^2^	–
	Aramid fiber/Nylon	Impact strength: 185 kJm^2^	–
[45]	Carbon fiber (41vol%)/Nylon	Elastic modulus: 13.0 GPa Flexural strength: 430 MPa Flexural modulus: 38.1 GPa	450
	Glass fiber (35vol%)/Nylon	Elastic modulus: 7.20 GPa Flexural strength: 149 MPa Flexural modulus: 14.7 GPa	600
[46]	Carbon fiber (3K)/Epoxy resin	Elastic modulus: 161 GPa Flexural strength: 202 MPa Flexural modulus: 144 GPa	793
[47]	Carbon fiber (48.7wt%)/Nylon	A 40% increase in flexural strength regarding chopped CFRP samples	271

**Table 2:** Summarized tensile test results.

Group	Type	Yield Strength [MPa]		Elasticity Modulus [GPa]		Ultimate Strain [%]	
		Value	Mean/CV [%]	Value	Mean/CV [%]	Value	Mean/CV [%]
Unreinforced (Section 2.1)	Reference Prusa	40.8	42.1/ 3.61	2.15	2.21/ 4.37	4.01	4.35/ 15.2
		42.6		2.28		4.48	
		41.3		2.24		4.24	
		44.6		2.33		5.37	
		40.5		2.06		3.39	
		42.5		2.21		4.60	
	Reference Inorega	44.2	42.1/ 4.95	2.32	2.23/ 4.83	3.91	4.16/ 5.81
		43.0		2.28		4.11	
		42.5		2.02		4.49	
		43.9		2.24		4.27	
		39.3		2.24		4.33	
		39.8		2.29		3.88	
	Recycled Inorega	43.8	43.9/ 1.26	2.32	2.35/ 3.05	3.92	4.47/ 8.79
		43.2		2.25		4.37	
		44.2		2.37		4.23	
		43.6		2.31		4.93	
		43.7		2.37		4.91	
		44.8		2.46		4.48	
Reinforced (Section 2.2)	Reference (unreinforced) Inorega	33.3	31.4/ 14.4	1.80	1.63/ 7.07	2.31	2.43/ 12.5
		28.6		1.63		2.17	
		38.4		1.63		2.95	
		27.5		1.47		2.34	
		29.0		1.62		2.37	
	Reinforced Inorega	47.3	50.8/ 5.12	1.93	1.66/ 22.0	2.77	3.13/ 13.5
		52.3		1.50		3.17	
		50.7		2.15		2.71	
		49.5		1.43		3.26	
		54.1		1.29		3.75	

## Data Availability

The authors will provide the raw data of this work upon request.
